# Commentary: Autoimmune/Autoinflammatory Syndrome Induced by Adjuvants (ASIA Syndrome) After Polypropylene Mesh Implantation – Protocol of a Pilot Study for Diagnostics and Treatment

**DOI:** 10.3389/jaws.2025.15402

**Published:** 2026-01-12

**Authors:** Nicholas T. H. Farr, Jan Willem Cohen Tervaert

**Affiliations:** 1 School of Chemical, Materials and Biological Engineering, University of Sheffield, Sheffield, United Kingdom; 2 Insigneo Institute for In Silico Medicine, University of Sheffield, Sheffield, United Kingdom; 3 Divsion of Rheumatology, Department of Medicine, University of Alberta, Edmonton, AB, Canada; 4 School for Mental Health and Neurosciences, Maastricht University, Maastricht, Netherlands

**Keywords:** autoimmune/autoinflammatory syndrome induced by adjuvants (ASIA), polypropylene mesh, mesh allergy testing, implant degradation, autoimmunity

We read with great interest the manuscript titled “Autoimmune/Autoinflammatory Syndrome Induced by Adjuvants (ASIA Syndrome) After Polypropylene Mesh Implantation–Protocol of a Pilot Study for Diagnostics and Treatment” [1]. In the proposed prospective Dutch study, the authors aim to include fifty patients who developed ASIA after polypropylene (PP) mesh placement. These patients will be followed for 1 year. Outcome measures include: mesh allergy, positive testing for (some) autoimmune disorders, and resolution of complaints after (partial) mesh removal.

Having recently authored a clinical outlook emphasising the need for studies investigating the potential relationship between PP mesh and ASIA [2] and the first clinical prospective study evaluating the presence of systemic effects after PP mesh placement [3], we were pleased to see this protocol and commend the authors for their initiative in this important area of research.

There are, however, several issues that should be discussed regarding the proposed study.

Firstly, the protocol appears to rest on the assumption that PP is inert within the human body, i.e., it does not change/degrade over time, which is incorrect [2]. Commercially available PP for medical purposes contains various additives such as antioxidants. Without such additives, PP rapidly loses its mechanical integrity due to oxidation, rendering it unsuitable for long-term weight-bearing applications such as soft tissue reinforcement in hernia repair [4], although additives never completely prevent degradation. Consequently, degradation-associated PP nanoparticles and microparticles could be quantified in the surrounding tissue after implantation, and an increased particle concentration over time was detected for a commercially available PP mesh [2]. The oxidative degradation of PP mesh has been extensively characterised *in vitro* and *in vivo* both chemically and biologically [2, 5]. Studies on explanted meshes from humans have consistently shown evidence of both bulk and surface degradation. While the bulk mechanical properties of PP may persist for a longer period, its surface chemistry, and hence its interactions with immune cells in the host tissue, often undergoes alterations much earlier [2, 5]. Such surface degradation changes both the mechanical properties and chemical composition of PP [6], which in turn modulates biological responses. For example, macrophages respond significantly differently when exposed to degraded versus pristine PP [7]. We therefore fear that the complex dynamic interplay between the immune system and the degrading PP over years with particle detachment *in vivo* cannot be mimicked with the proposed mesh allergy testing (MAT) method.

Additionally, the authors state: “*For the MAT, 1 mL methyl ethyl ketone (MEK) is used to dissolve a 5 cm* ^*2*^ *mesh for at least 48 hours, up to a maximum of 72 hours.”* While this description suggests complete dissolution, MEK cannot, in fact, dissolve PP, which is a non-polar semicrystalline polymer typically soluble only in non-polar solvents such as xylene. It is therefore likely that the authors intended to use MEK as an extractant rather than a solvent, to leach low-molecular-weight additives or surface residues from the mesh. This distinction is important, because such extraction would not reproduce the *in vivo* degradation processes (oxidation, particle detachment) that occur over time. Accordingly, the proposed MAT may primarily reflect acute responses to soluble additives rather than chronic immune activation driven by polymer degradation. Given the time-dependent nature of PP degradation [2, 5], the proposed follow-up period appears insufficient to capture the full spectrum of potential outcomes. This concern is further supported by our findings, which show that around 40% of patients developed ASIA symptoms more than 1 year after implantation [3]. It is also important to note that PP meshes from different manufacturers have different degradation behaviour and chemical composition due to their varying formulations, i.e., supplementation with different additives in different concentrations [2, 5]. Therefore, care must be taken in making generalised statements based on results derived from a limited number of products/manufacturers.

Secondly, the protocol for autoimmune testing is rather limited. Whereas, C-reactive protein levels in patients with ASIA due to mesh are, generally, in the normal range, angiotensin-converting enzyme and soluble interleukin-2 receptor levels are often elevated [8]. Importantly, in the proposed protocol these markers are not evaluated.

Thirdly, the surgical procedure for (partial) mesh removal is not well described. It should be emphasised that previous studies demonstrated that only complete mesh removal resulted in amelioration of systemic symptoms [3, 9], whereas partial removal may result in a decrease of pain but not in a decrease of systemic symptoms [3]. Unfortunately, complete removal of PP mesh is often not feasible in anatomically constrained regions such as the groin, where space is limited and nerves are present. Moreover, due to particle detachment and migration, residual PP fragments may continue to stimulate the immune system and cause systemic symptoms even after ‘complete’ mesh removal.

Fourthly, the authors state that there is no solid evidence that ASIA is caused by PP mesh [1]. To determine whether an association exists between a triggering event and outcome, scientists currently consider an evaluation using the so-called Bradford Hill criteria [10]. Based on these criteria, we suggest that an association between PP mesh and ASIA is likely [8].

We appreciate the initiative to perform a prospective study on ASIA due to mesh and hope that our concerns will be considered to strengthen the scientific rigor and clinical relevance of the work. We look forward to following the results of this important study.
